# Effectiveness of Meniscus Root Tear Repair Versus Conservative Therapy and Adjunct Therapies: A Systematic Review

**DOI:** 10.7759/cureus.75645

**Published:** 2024-12-13

**Authors:** Masateru Hayashi, Yuichi Isaji, Yasuyuki Kurasawa, Takashi Kitagawa

**Affiliations:** 1 Department of Rehabilitation, Matsuoka Orthopedic and Internal Medicine Rehabilitation, Gifu, JPN; 2 Department of Physical Therapy, School of Health Sciences, Bukkyo University, Kyoto, JPN; 3 Department of Rehabilitation, Faculty of Health Sciences, Nagano University of Health and Medicine, Nagano, JPN; 4 Department of Physical Therapy, School of Health Sciences, Shinshu University, Matsumoto, JPN

**Keywords:** activities of daily living, conservative therapy, knee joint, meniscus root tears, quality of life

## Abstract

The meniscus plays a vital role in knee biomechanics, particularly in load distribution and stability. Meniscus root tears (MRTs) compromise these functions, resulting in biomechanical alterations and knee osteoarthritis. The effectiveness of different MRT treatments is not yet well defined. This study aimed to compare the effectiveness of MRT repair versus conservative therapy and conservative therapy versus multimodal or adjunct therapy in patients with MRTs. A systematic review of clinical studies was performed using electronic searches of PubMed, CENTRAL, CINAHL, and PEDro databases. Two reviewers independently conducted screening, data extraction, risk of bias assessment, and the Grading of Recommendations Assessment, Development, and Evaluation (GRADE). The database search identified 3,837 articles, of which 11 (three randomized controlled trials [RCTs] and eight cohort studies) were relevant. MRT repair surgery and conservative treatment improved the Knee Injury and Osteoarthritis Outcome Score (KOOS) subscales for activities of daily living (ADL) and quality of life (QOL). Multimodal or adjunct therapies also enhanced KOOS subscales for ADL and QOL. In the bias risk assessment, three RCTs were of high risk, five cohort studies were of high quality, and three were of moderate quality. Additionally, the certainty of evidence was very low for all comparisons in GRADE. Therefore, the effectiveness of MRT repair, conservative therapy, and multimodal therapy remains unclear owing to the risk of bias and low certainty of evidence. Whether MRT repair versus conservative therapy and conventional versus multimodal or adjunct therapy are more effective in improving ADL and QOL remains unclear. Future treatment decisions require long-term, high-quality research including controlled and randomized trials with large sample sizes.

## Introduction and background

The meniscus plays an essential role in the normal knee biomechanics. This contributes to load transfer and joint stability [1]. Additionally, the collagen fibers and root of the meniscus must remain intact to distribute the load on the knee [2]. Meniscus root tears (MRTs) result in reduced function of the circumferential fibers of the knee and reduced meniscal ability to withstand hoop stress in the medial compartment of the knee [3-4]. MRTs can alter the biomechanics of the knee joint and may cause progressive cartilage damage, osteoarthritis (OA), and other problems [2,5]. Additionally, meniscal extrusion occurs within a short period after MRTs and progresses to OA of the knee at an early stage [6]. Therefore, treatment of MRTs remains essential.

MRT treatments can be categorized into two groups: conservative therapy, which includes physical therapy, medication, and joint injections, and surgical therapy, which encompasses partial meniscectomy and surgical repair [7]. Indications for treatment are determined by the type and extent of the tear, indications and contraindications for surgical repair, and cartilage condition [8]. It has been suggested that MRT repair is more cost-effective than meniscectomy or conservative therapy [9] and may delay the transition to knee OA or total knee arthroplasty (TKA) [10]. Conservative therapy is intended to relieve symptoms, and comparisons between conventional physical therapy (exercise therapy) and multimodal therapy (conventional physical therapy plus manual therapy) show the effectiveness of multimodal therapy [4,11,12]. Several comparative studies have yielded conflicting results regarding the relative effectiveness of nonoperative, operative, and repair treatment options based on general outcome scores [13-16]. Although many studies report the therapeutic effects of MRTs, it remains unclear whether surgical or conservative therapy is more effective, and the quality of both studies is not assured [1,4]. To date, no randomized controlled trials (RCT) comparing surgical and conservative therapy outcomes have been reported in systematic reviews. In addition, the limitations reported in previous studies include selection bias due to small sample sizes, heterogeneity of results, and low levels of evidence (LoEs) of the included studies [1,17]. In recent years, RCTs on conservative therapy for patients with MRTs have been reported [12], which may help overcome the limitations of previous systematic reviews. Therefore, it is necessary to re-examine the effectiveness of treatment in patients with MRTs. This study had two aims: first, to determine the effectiveness of MRT repair versus conservative therapy for patients with MRTs, and second, to assess the effectiveness of conventional versus multimodal or adjunctive therapies in these patients.

## Review

Methods

This systematic review was conducted in accordance with the Preferred Reporting Items for Systematic Reviews and Meta-Analyses [18]. The protocol for this review was predetermined by the review team and registered in PROSPERO (ID: CRD42023414833; https://www.crd.york.ac.uk/PROSPERO/display_record.php?RecordID=414833).

Search Strategy

Various electronic bibliographic databases were used to search for appropriate literature. These databases included PubMed, the Cochrane Central Register of Controlled Trials via Ovid, the Cumulative Index to Nursing and Allied Health Literature via EBSCO, and the Physiotherapy Evidence Database. Moreover, a search for grey literature was conducted using the OpenGrey platform and ClinicalTrials.gov. The search criteria encompassed the following keywords: "meniscal root tears", "physical therapy", and "meniscal root tears repair" (Table 1). The search was conducted from the establishment of the database until April 22, 2023, without language restrictions.

**Table 1:** Database search strategies

Database	Search strategy
PubMed	("Meniscus"[MeSH Terms] OR "meniscu*"[Title/Abstract] OR "Tibial Meniscus Injuries"[MeSH Terms] OR "Tibial Meniscus Injuries"[Title/Abstract] OR "medial menisc*"[Title/Abstract] OR "root tear*"[Title/Abstract] OR "posterior root tear*"[Title/Abstract] OR "posterior horn tear*"[Title/Abstract] OR "horn tear*"[Title/Abstract]) AND ("Physical Therapy Modalities"[MeSH Terms] OR "physical therapy"[Title/Abstract] OR "nonsurg*"[Title/Abstract] OR "nonoperat*"[Title/Abstract] OR "conserv*"[Title/Abstract] OR "rehab*"[Title/Abstract] OR "brace*"[Title/Abstract] OR "exercis*"[Title/Abstract] OR "manual therapy"[Title/Abstract] OR "aerobic exercis*"[Title/Abstract] OR "cast*"[Title/Abstract] OR "Debridement"[MeSH Terms] OR "debride*"[Title/Abstract] OR "repair*"[Title/Abstract] OR "Arthroscopy"[MeSH Terms] OR "arthroscop*"[Title/Abstract] OR "meniscectomy"[Title/Abstract] OR "pharmacotherapy"[Title/Abstract]) AND (("randomized controlled trial"[Publication Type] OR "controlled clinical trial"[Publication Type] OR ("randomized"[Title/Abstract] OR "randomized"[Title/Abstract]) OR "placebo"[Title/Abstract] OR "drug therapy"[MeSH Subheading] OR "randomly"[Title/Abstract] OR "trial"[Title/Abstract] OR "groups"[Title/Abstract] OR "Cohort Studies"[MeSH Terms]) NOT ("Animals"[MeSH Terms] NOT "Humans"[MeSH Terms]))
CENTRAL	([exp meniscus] OR [meniscu*.tw.] OR [exp "tibial meniscus injuries"] OR ["tibial meniscus injuries".tw.] OR ["medial menisc*".tw.] OR ["root tear*".tw.] OR ["posterior root tear*".tw.] OR ["posterior horn tear*".tw.] OR ["horn tear*".tw.]) AND ([exp "physical therapy modalities"] OR ["physical therapy".tw.] OR [nonsurg*.tw.] OR [nonoperat*.tw.] OR [conserv*.tw.] OR [rehab*.tw.] OR [brace*.tw.] OR [exercis*.tw.] OR ["manual therapy".tw.] OR ["aerobic exercis*".tw.] OR [cast*.tw.] OR [exp debridement] OR [debride*.tw.] OR [repair*.tw.] OR [exp arthroscopy] OR [arthroscop*.tw.] OR [meniscectomy.tw.] OR [pharmacotherapy.tw.])
CINAHL	((MH meniscus+) OR (TI meniscu* OR AB meniscu*) OR (MH "tibial meniscus injuries+") OR (TI "tibial meniscus injuries" OR AB "tibial meniscus injuries") OR (TI "medial menisc*" OR AB "medial menisc*") OR (TI "root tear*" OR AB "root tear*") OR (TI "posterior root tear*" OR AB "posterior root tear*") OR (TI "posterior horn tear*" OR AB "posterior horn tear*") OR (TI "horn tear*" OR AB "horn tear*")) AND ((MH "physical therapy modalities+") OR (TI "Physical Therapy" OR AB "Physical Therapy") OR (TI nonsurg* OR AB nonsurg*) OR (TI nonoperat* OR AB nonoperat*) OR (TI conserv* OR AB conserv*) OR (TI rehab* OR AB rehab*) OR (TI brace* OR AB brace*) OR (TI exercis* OR AB exercis*) OR (TI "manual therapy" OR AB "manual therapy") OR (TI "aerobic exercis*" OR AB "aerobic exercis*") OR (TI cast* OR AB cast*) OR (MH debridement+) OR (TI debride* OR AB debride*) OR (TI repair* OR AB repair*) OR (MH arthroscopy+) OR (TI arthroscop* OR AB arthroscop*) OR (TI meniscectomy OR AB meniscectomy) OR (TI pharmacotherapy OR AB pharmacotherapy)) AND ((PT "randomized controlled trial")) OR ((PT "controlled clinical trial")) OR ((TI randomized OR AB randomized) OR (TI randomised OR AB randomised)) OR ((TI placebo OR AB placebo)) OR ("drug therapy") OR ((TI randomly OR AB randomly)) OR ((TI trial OR AB trial)) OR ((TI groups OR AB groups)) OR (MH "cohort studies+")) NOT ((MH animals+) NOT (MH humans+))
PEDro	Abstract & Title: menisc* Therapy: Fitness training OR Strength training Body Part: Lower leg OR knee
OpenGrey	menisc*
ClinicalTrials.gov	Meniscus Root Tear

Eligibility Criteria

This systematic review was to examine studies that met the following criteria:

(i) Study participants afflicted with MRTs (medial or lateral), encompassing Kellgren and Lawrence (KL) classification, Ahlback classification, any age, and both genders.

(ii) Surgical and conservative therapies included MRT repair and conservative therapies (medication [simple and nonsteroidal anti-inflammatory analgesics]), rehabilitation, physical therapy, exercise, manual therapy, orthotic intervention, medication administration, aerobic exercise program, and injection therapy (including glucocorticoid injection, multi-platelet plasma injection, stem cell therapy, and cell therapy). In conventional and multimodal therapies, multimodal therapy includes manual rehabilitation in addition to exercise therapy.

(iii) The principal outcome measures encompassed activities of daily living (ADL) and quality of life (QOL), with secondary outcomes including pain, physical functionality, body mass index (BMI), KL, and Ahlback classifications. Outcomes were assessed using the Western Ontario and McMaster Universities Arthritis Index, Tegner activity score, International Knee Documentation Committee (IKDC) score, Lysholm Knee Scoring Scale, Knee injury and Osteoarthritis Outcome Score (KOOS), Western Ontario Meniscal Evaluation Tool, KL classification, Ahlback classification, 36-item Short Form Health Survey, and Euro QoL 5-dimension instruments. Adverse events of interest included incidents resulting in mortality (e.g., deep vein thrombosis, cardiovascular events, and pulmonary events), pain exacerbations, and falls.

(iv) Study designs included RCTs, quasi-RCTs, and cohort studies (including prospective and retrospective designs).

The exclusion criteria were as follows: the cases were defined as OA of the knee only, cartilage degeneration only, surgery other than MRTs repair, and meniscus injury with ligament rupture.

Study Selection

Screening activities were executed by two independent reviewers (M.H. and Y.I.) who meticulously scrutinized titles, abstracts, and full texts against pre-established eligibility criteria using the Rayyan web application [19]. In the case of discrepancies in screening decisions, the decision was made in consultation with a third reviewer (T.K.).

Data Extraction

The following data were extracted: author, year of publication, study design, abstract, aim, study participants (characteristics and sample size), setting or environment, description of the intervention, description of the control group (surgical procedure), follow-up period, outcome score, adverse events, funding source, extraction results, primary results of the review (relevant to this review), and limitations and conclusions (clinical and research significance). Regarding outcome metrics, post-intervention values or change scores were extracted when accessible. Data extraction was conducted independently by two reviewers (M.H. and Y.I.). In cases of discordance between the two evaluators, a third reviewer (T.K.) was consulted for adjudication. Furthermore, when essential data were absent from the incorporated articles, efforts were made to engage the respective study authors for data acquisition.

Strategy for Data Synthesis

A meta-analysis was conducted when feasible by calculating the continuous variables of ADL and QOL using the mean difference (MD) or standardized MD, along with 95% confidence intervals (CIs), if there were two or more RCTs. Additionally, dichotomized data were analyzed as risk ratios with a 95% CI. The integration of means and standard deviations for continuous variables followed the methodology outlined in the Cochrane Handbook [20]. Furthermore, owing to the expected heterogeneity across studies, such as differences in sex, age, and country, a random-effects model was employed. Heterogeneity was analyzed to investigate homogeneity among the reviewed studies. To check for heterogeneity among studies, we used the Q statistic and I², where I² was greater than 50%, and a significant Q statistic (p = 0.05) suggested the inclusion of studies with heterogeneity. Review Manager software (RevMan 5.4) was used for the statistical analysis. If the included studies exhibited heterogeneity, small sample sizes, methodological differences, and differences in outcomes across studies, it was recommended that data pooling be refrained, as these factors led to unreliable outcome estimates [20]. Therefore, meta-analyses were not performed if applicable.

Quality Assessment and Quality of Evidence

The methodological quality of the included RCTs was assessed using the Cochrane Collaboration's Risk of Bias 2 tool [21]. This assessment encompassed the following domains: (i) bias arising from the randomization process, (ii) bias stemming from deviations in adherence to intended interventions, (iii) bias resulting from missing outcome data, (iv) bias in outcome measurement, and (v) bias associated with the selection of reported results. Each study was appraised as having a low risk of bias, harboring some concerns of bias, or exhibiting a high risk of bias. Non-randomized studies were evaluated using the Newcastle-Ottawa Scale (NOS), a dependable quality appraisal instrument for nonrandomized cohort studies (Appendix A) [22]. The NOS comprises eight items categorized into three domains: selection, comparability, and outcomes, with ratings allocated using a star-based system ranging from 0 (indicative of low quality [LQ]) to 9 (reflecting high quality [HQ]) points. An evaluation with a score of 0 to 3 points was considered LQ, an evaluation with a score of 4 to 6 points was considered moderate quality (MQ), and an evaluation with a score of 7 to 8 points was considered HQ [23,24]. Both reviewers (M.H. and Y.I.) independently evaluated the studies. In case of disagreement, a third reviewer (T.K.) was consulted.

Appraisal of the quality of evidence for the summary of findings was executed using the Grading of Recommendations Assessment, Development, and Evaluation (GRADE) approach [25]. This process was conducted using GRADEpro software (http://www.guidelinedevelopment.org/) to generate a summary table of findings regarding ADLs, QOL, and adverse treatment outcomes. The quality of the evidence was assessed by one author (M.H.), consulted by two others (Y.K. and T.K.), and finalized.

Results

Search Results

**Figure 1:**
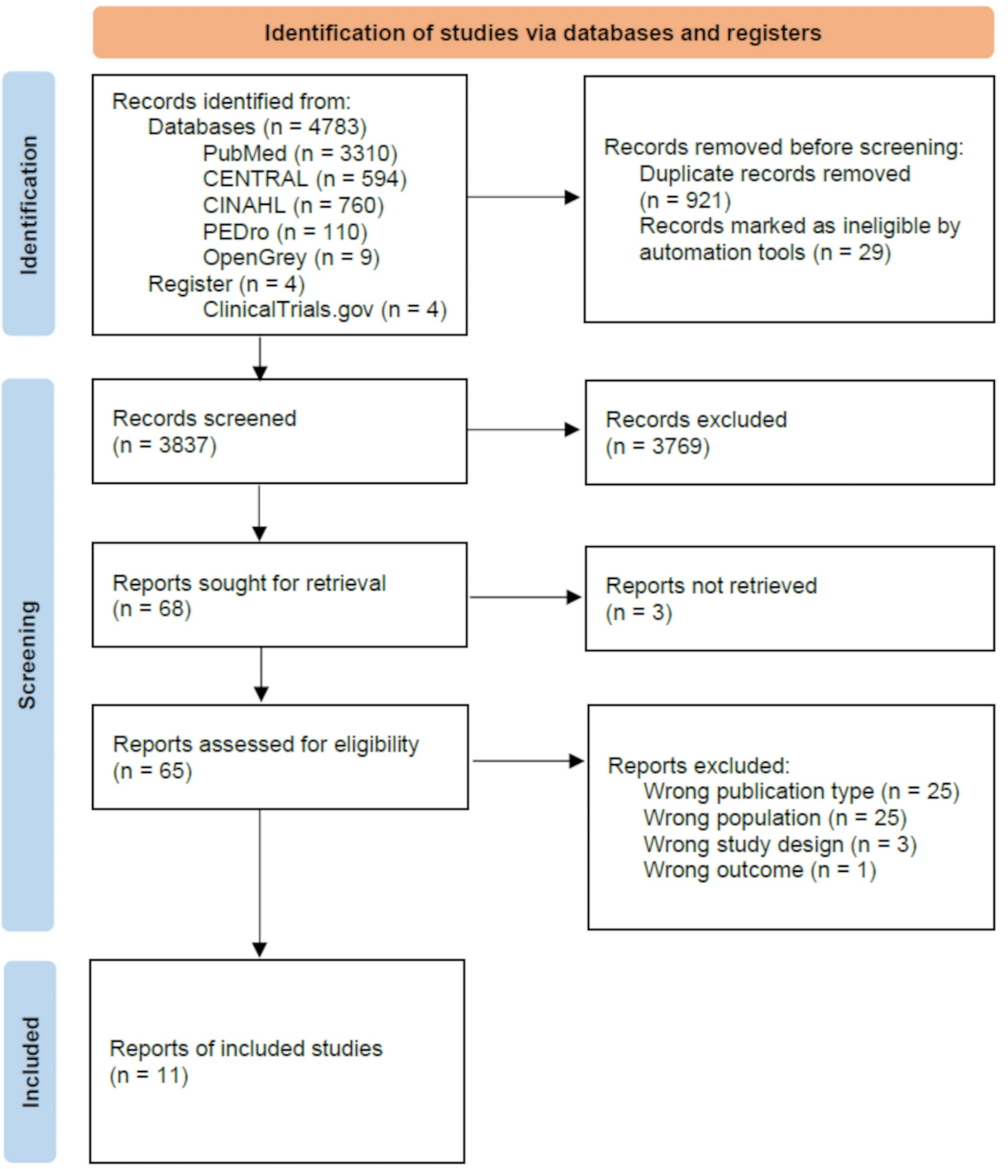
PRISMA flow diagram showing the results of the database search and screening PRISMA, Preferred Reporting Items for Systematic Reviews and Meta-Analyses

The search identified 3,837 articles, of which 3,769 were excluded through title and abstract screening, and 65 were subjected to full-text screening. Finally, 11 studies were included [12,26-35]. The study designs of the 11 applicable papers included three RCTs [12,26,27] and eight cohort studies [28-35]. The screening procedure and rationale for exclusion are shown in the flowchart in Figure 1.

Characteristics of Included Studies

The characteristics of the studies included in this systematic review are presented in Table 2. Four studies compared the restorative effects of MRTs versus conservative treatment approaches [28-31]. In addition, three cohort studies compared MRT repair with conventional physical therapy [28-30] and one cohort study focused on the rehabilitation of MRT repair [31]. LoEs were 2B, 2C, and 3B [36]. Conventional therapy versus multimodal therapy or adjunct therapy (conventional physical therapy plus electrical muscle stimulation or electromyographic biofeedback) were compared in seven studies [12,26,27,32-35].

**Table 2:** Characteristics of included studies *Intervention/control AAE, adapting alignment exercise; AMR, arthroscopic meniscal repair; BMI, body mass index; EMG, electromyographic; EMG-BFB, electromyographic biofeedback; HHD, hand-held dynamometry; IKDC, International Knee Documentation Committee; JKOM, Japanese Knee Osteoarthritis Measure; JLCA, joint line convergence angle; JOA, Japanese Orthopedic Association knee rating score; KL, Kellgren and Lawrence; KOOS, Knee injury and Osteoarthritis Outcome Score; LLERT, lower limb exoskeleton robot training; LoE, level of evidence; Lysholm, Lysholm knee scoring scale; MCS, mental component summary scores; MRTs, meniscus root tears; MTE, muscle training and exercise; NA, not applicable; NRS, numerical rating scale; PCS, physical component summary; PRP, platelet-rich plasma; RCT, randomized controlled trial; ROM, range of motion; SD, standard deviation; Tegner, Tegner activity scale; VAS, visual analog scale

Author, year	Study design (LoE)	Participants			Intervention			Control			Outcomes	Follow-up, time points (SD)
		No. of patients*	Meniscus repair	KL	Age (SD/range)	BMI (SD)	Methods	Age (SD)	BMI (SD)	Methods		
MRTs repair versus conservative therapy												
Ahn et al., 2015 [28]	Retrospective cohort study (2B)	13/25	AMR	1-4	62.0 (10.5)	26.4 (4.0)	Conservative treatment	56.0 (8.0)	25.1 (3.8)	AMR	IKDC, Tegner and Lysholm	Mean: 17.4 (7.8) months
Dragoo et al., 2020 [29]	Cohort study (2B)	18/30	AMR	1-3	64.8 (9.0)	28.8 (4.6)	Conservative treatment	56.7 (11.1)	29.0 (6.7)	AMR	KOOS, Lysholm, Tegner, PCS, MCS	Mean: 4.4 years
Kwak et al., 2018 [30]	Retrospective cohort study (2C)	57/31	AMR	<3	60.0 (7.4)	25.4 (4.0)	Conservative treatment	59.7 (7.2)	24.7 (4.6)	AMR	JLCA, KL	3 months
Tahami et al., 2022 [31]	Prospective cohort study (3B)	43	AMR	<2	53.2 (8.1)	28.1 (2.0)	Postoperative rehabilitation	NA	NA	NA	Lysholm	2 years
Conventional therapy versus multimodal therapy (conventional physical therapy plus manual therapy) or adjunct therapy (conventional physical therapy plus electrical muscle stimulation/electromyographic biofeedback)												
Ikuta et al., 2020 [12]	RCT (1C)	13/13	NA	<2	67.9 (7.2)	22.9 (2.3)	AAE	68.2 (10.8)	24.2 (2.3)	MTE	VAS, JOA, JKOM, KOOS, Tegner	6 months
Oravitan and Avram, 2013 [26]	RCT (1C)	33/31	AMR	NA	33.2 (6.5)	NA	EMG-BFB and rehabilitation	32.5 (5.8)	NA	Rehabilitation	EMG, HHD, KOOS	1 week, and 2 months
Wang et al., 2022 [27]	RCT (1C)	45/45	AMR	NA	40.1 (12.3)	NA	Conventional rehabilitation and LLERT	41.4 (12.6)	NA	Conventional rehabilitation	IKDC, Lysholm, ROM	2 days, and 2 months
Krych et al., 2017 [32]	Retrospective cohort study (3B)	52	NA	NA	58.0 (10.0)	NA	Physical therapy	NA	NA	NA	IKDC, Tegner, KL	2 years
Lim et al., 2010 [33]	Retrospective cohort study (3B)	30	NA	<2	59.0 (51 to 65)	NA	Pharmacotherapy and physical therapy	NA	NA	NA	VAS, Lysholm, IKDC	6, 12 months, and final follow-up
Neogi et al., 2013 [34]	Prospective cohort study (3B)	37	NA	1 to 2	55.8 (50 to 62)	NA	Exercise therapy	NA	NA	NA	VAS, Lysholm, Tegner, KL	3, 6, 12, and 24 months
Popescu et al., 2020 [35]	Retrospective cohort study (3B)	30	NA	NA	13.9 (1.4)	NA	PRP	NA	NA	NA	NRS, Lysholm	3 months

There was one RCT that compared conventional and multimodal therapy [12]. Two RCTs compared conventional and adjunct therapy [26,27]. Additionally, there was also one cohort study each on physical therapy, pharmacotherapy supplemented by physical therapy, exercise therapy, and multi-platelet plasma therapy [32-35]. The LoEs were 1C and 3B. Diverse outcome measures, including KOOS, Tegner activity score, IKDC, Lysholm knee scoring scale, KL classification, and visual analog scale (VAS), were evaluated across these studies. Many studies have evaluated multiple outcomes, and there are variations among studies. Diverse temporal intervals were used during follow-up. The RCTs were characterized by short-term durations, ranging from one week to two months. Conversely, cohort studies encompassed a spectrum of outcomes evaluated over both short and long durations, ranging from three months to approximately four years.

Quality Assessment of the Included Studies

**Figure 2:**
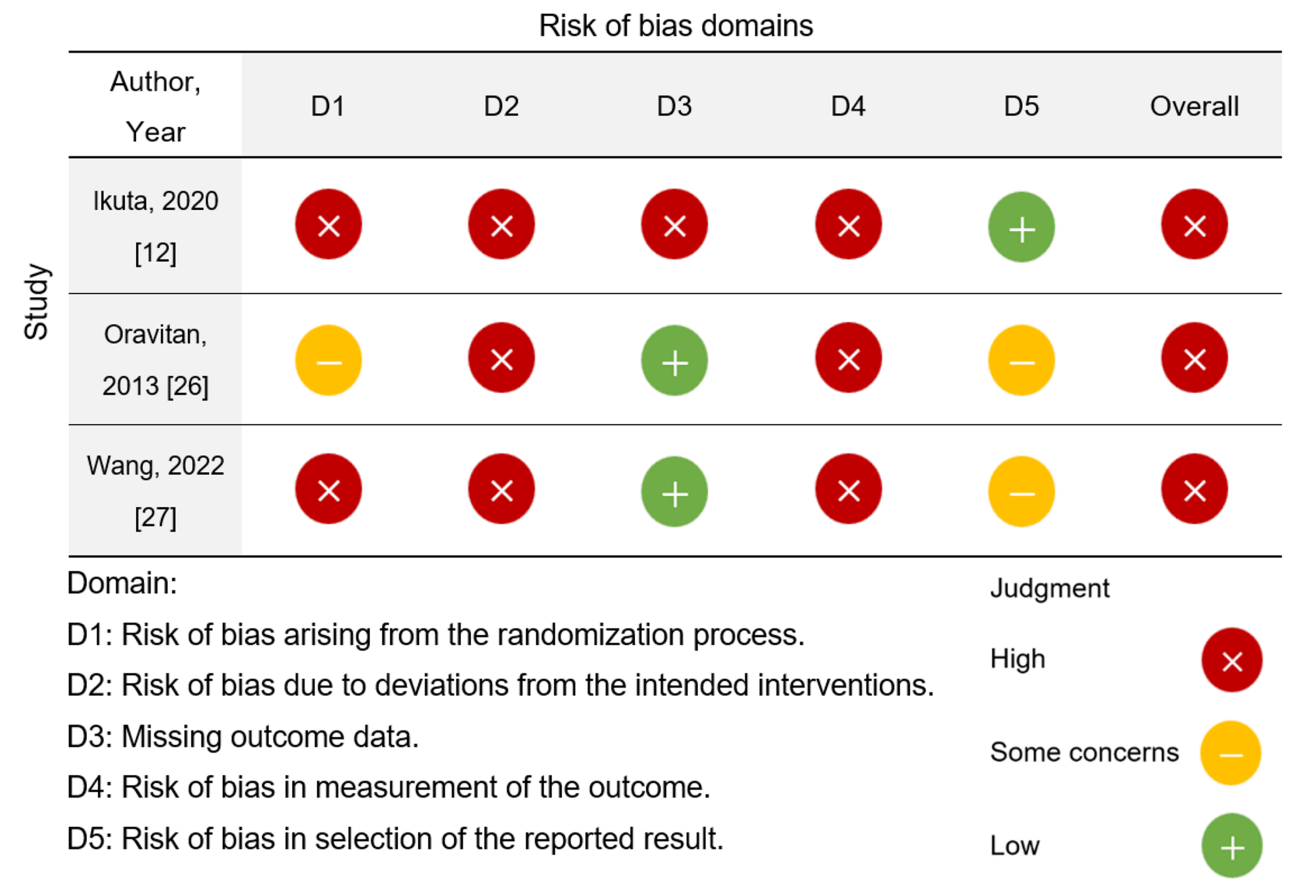
Assessing risk of bias in the included studies

A bias assessment was conducted for the three RCTs, which revealed a high risk of bias across all studies (Figure 2) [12,26,27]. Conversely, regarding the risk of bias in the eight cohort studies, the study quality was HQ in five studies [28,29,31,33,34] and MQ in three studies (Table 3) [30,32,35].

**Table 3:** The Newcastle-Ottawa Scale score of the included cohort studies 1: Representativeness of the exposed cohort (maximum: ★) 2: Selection of the non-exposed cohort (maximum: ★) 3: Ascertainment of exposure (maximum: ★) 4: Demonstration that outcome of interest was not present at the start of study (Maximum: ★) 5: Comparability of cohorts on the basis of the design or analysis (maximum: ★★) 6: Assessment of outcome (maximum: ★) 7: Was follow-up long enough for outcomes to occur (maximum: ★) 8: Adequacy of follow-up of cohorts (maximum: ★)

Author, year	Selection				Comparability	Outcome			Total score (out of 9)	Study quality
	1	2	3	4	5	6	7	8		
Ahn et al., 2015 [28]	★	★	★		★★	★		★	7/9	High
Dragoo et al., 2020 [29]	★	★	★		★★	★	★	★	8/9	High
Kwak et al., 2018 [30]	★	★	★		★★	★			6/9	Moderate
Tahami et al., 2022 [31]	★		★		★★	★	★	★	7/9	High
Krych et al., 2017 [32]	★		★		★★	★	★		6/9	Moderate
Lim et al., 2010 [33]	★		★		★★	★	★	★	7/9	High
Neogi et al., 2013 [34]	★		★		★★	★	★	★	7/9	High
Popescu et al., 2020 [35]			★		★	★		★	4/9	Moderate

Changes in Outcomes Between MRT Repair and Conservative Therapy

**Figure 3:**
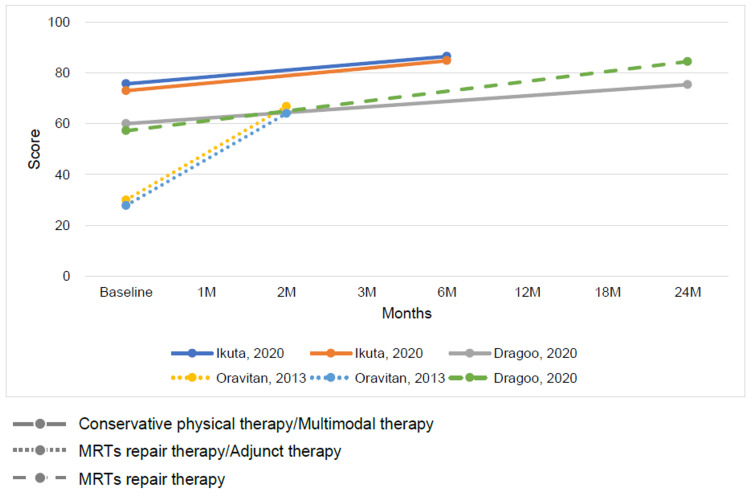
Change in mean Knee injury and Osteoarthritis Outcome Score subscale activities of daily living function score at baseline and follow-up for conservative physical therapy or multimodal therapy or adjunct therapy and meniscus root tear repair therapy [12,26,29]

**Figure 4:**
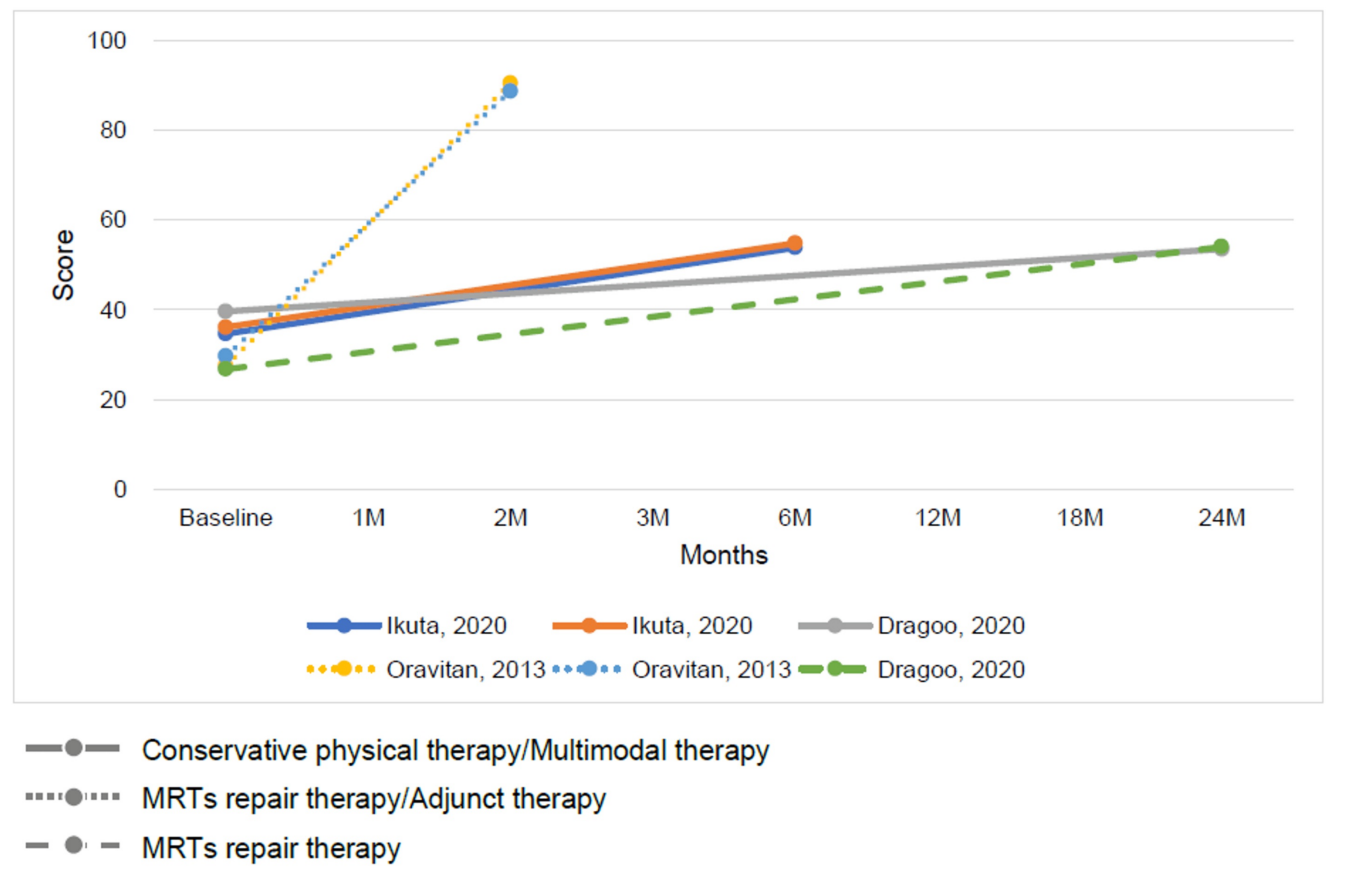
Change in mean Knee injury and Osteoarthritis Outcome Score subscale quality of life score at baseline and follow-up for conservative physical therapy or multimodal therapy or adjunct therapy and meniscus root tear repair therapy [12,26,29]

**Figure 5:**
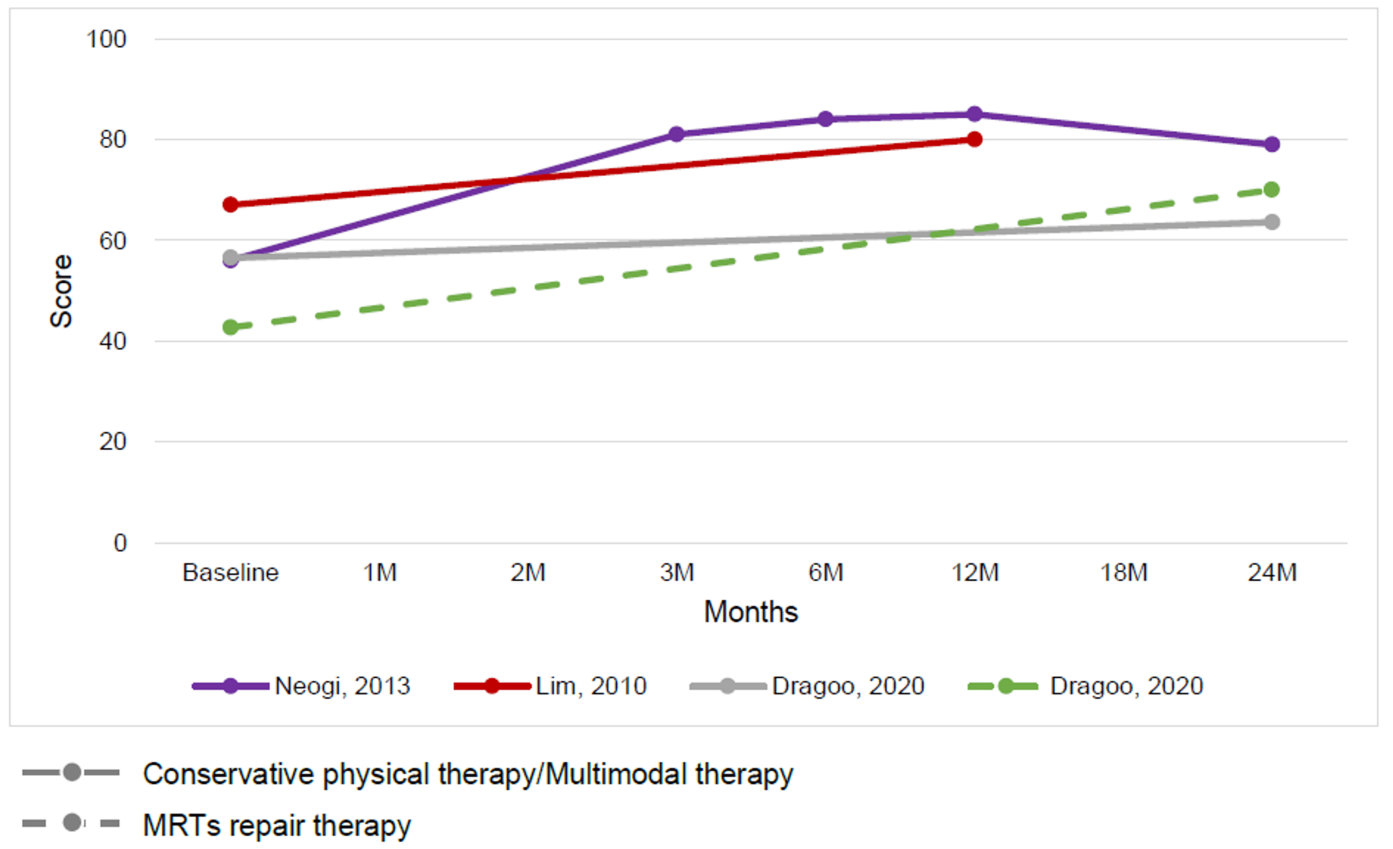
Change in mean Lysholm knee scoring scale at baseline and follow-up for conservative physical therapy or multimodal therapy and meniscus root tear repair therapy [29,33,34]

**Figure 6:**
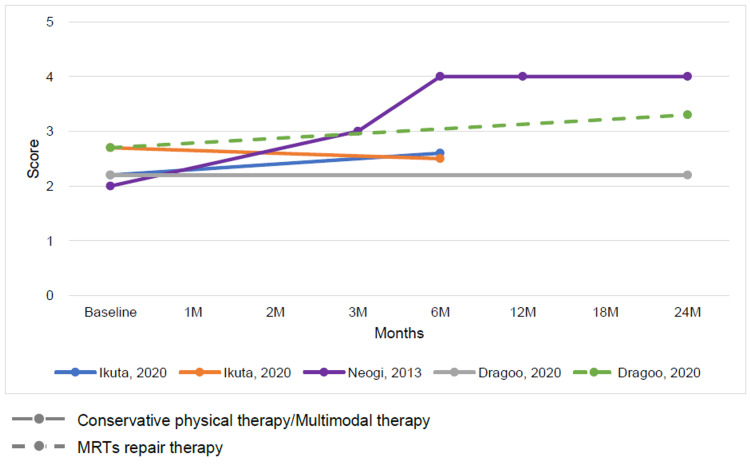
Change in mean Tegner activity score at baseline and follow-up for conservative physical therapy or multimodal therapy and meniscus root tear repair therapy [12,29,34]

The outcomes for MRT repair and conservative therapy were summarized by time point. The time points ranged from baseline to 24 months, with outcomes indicating a trend toward improved ADL function and QOL on the KOOS subscale for both MRT repair and conservative therapy (Figures 3, 4). The Lysholm knee scoring scale and Tegner activity score showed that conservative therapy had a short-term benefit but a plateau in long-term outcomes. In contrast, MRT repair showed a trend toward long-term improvement (Figures 5, 6).

Change in Outcomes Between Multimodal or Adjunctive Therapy and Conventional Physical Therapy

**Figure 7:**
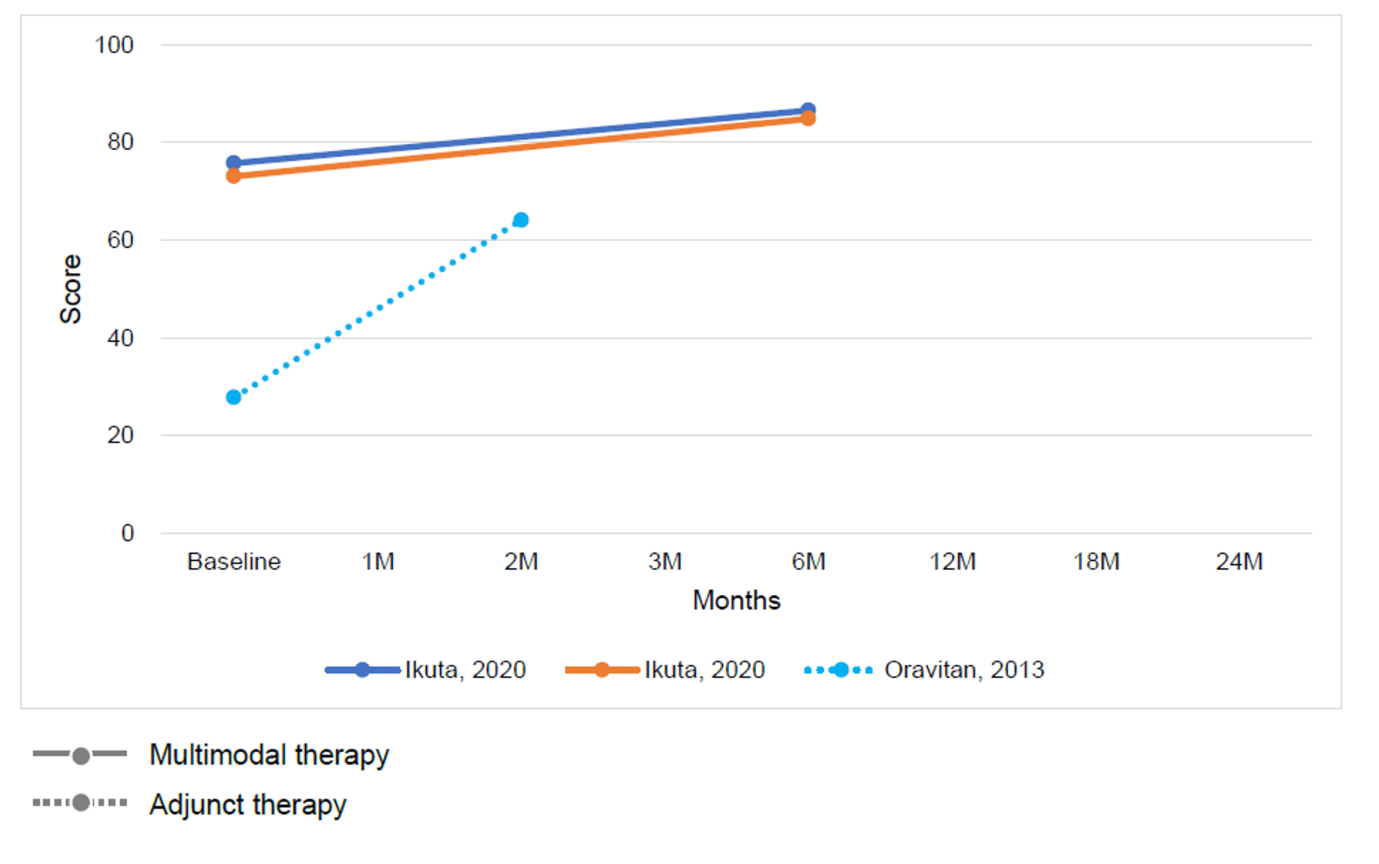
Change in mean Knee injury and Osteoarthritis Outcome Score subscale activities of daily living function score at baseline and follow-up for multimodal therapy or adjunct therapy [12,26]

**Figure 8:**
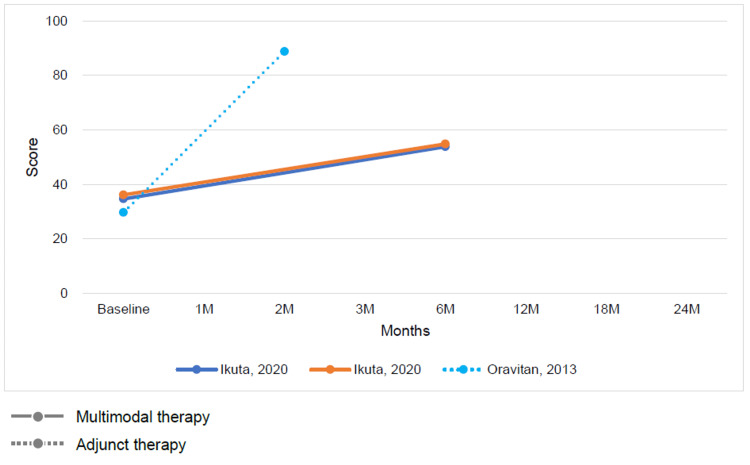
Change in mean Knee injury and Osteoarthritis Outcome Score subscale quality of life score at baseline and follow-up for multimodal therapy or adjunct therapy [12,26]

**Figure 9:**
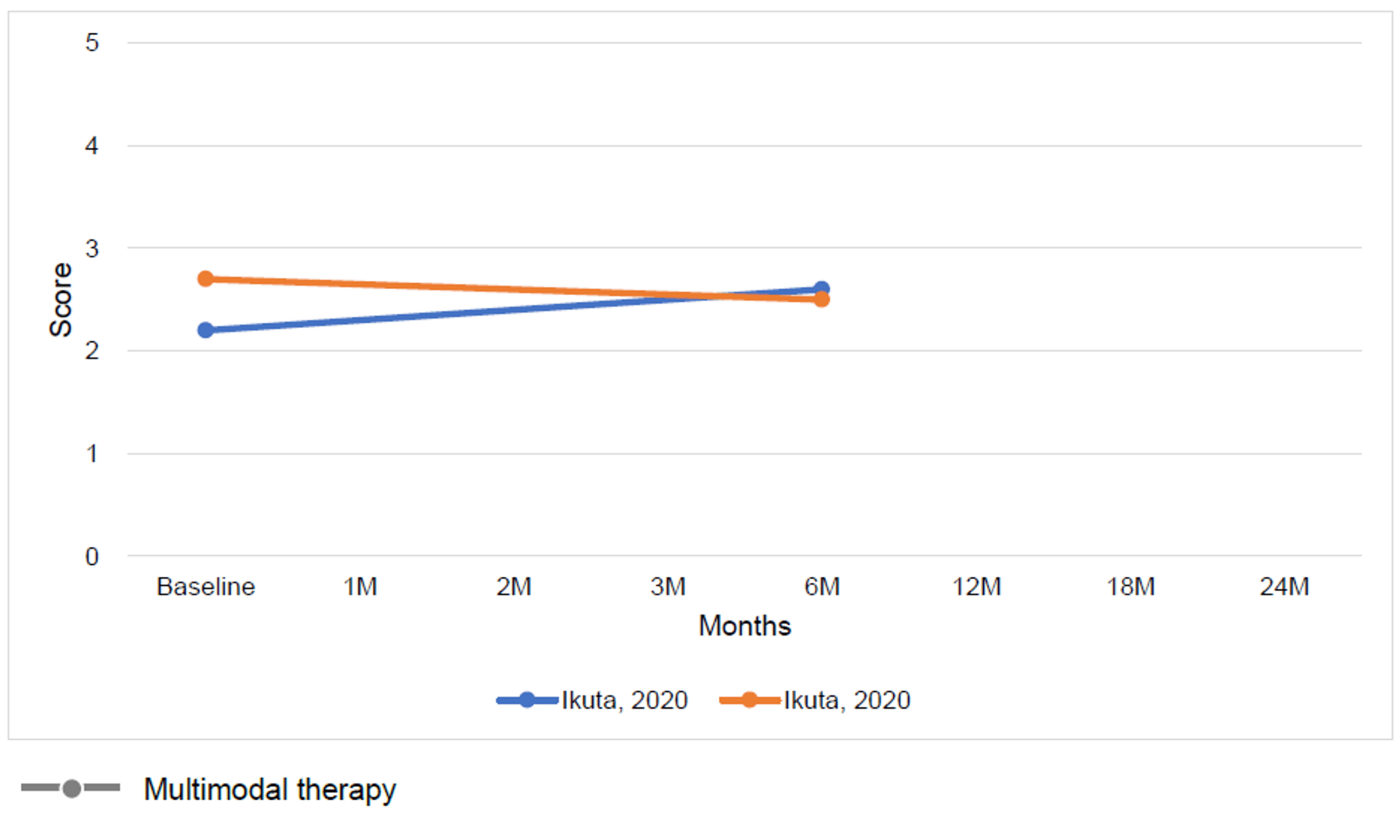
Change in mean Tegner activity score at baseline and follow-up for multimodal therapy [12]

The results of conventional therapy and multimodal or adjunct therapy, assessed at varying intervals ranging from baseline to 6 months, demonstrated improvements in both ADL function and QOL on the KOOS subscale for both therapy types (Figures 7, 8). Conversely, the Tegner activity score showed no characteristic changes (Figure 9).

Meta-analyses

**Figure 10:**
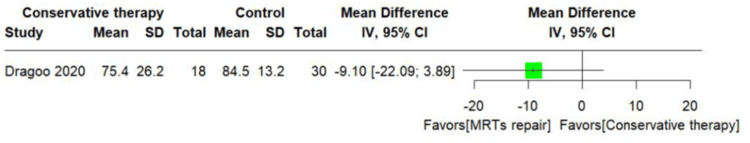
Change in mean Knee injury and Osteoarthritis Outcome Score subscale activities of daily living function score [29]

**Figure 11:**
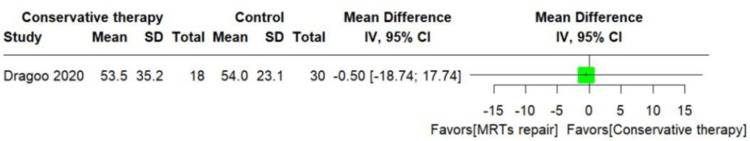
Change in mean Knee injury and Osteoarthritis Outcome Score subscale quality of life score [29]

**Figure 12:**
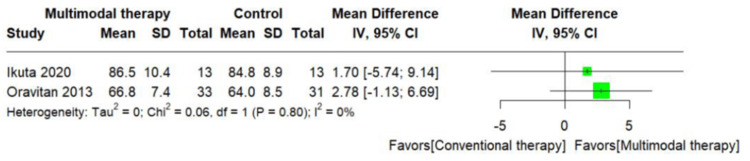
Change in mean Knee injury and Osteoarthritis Outcome Score subscale activities of daily living function score [12,26]

**Figure 13:**
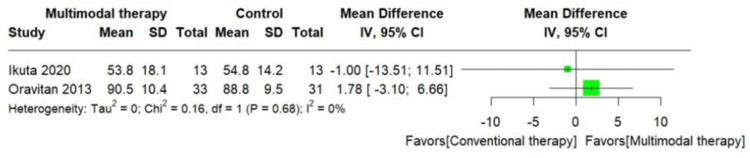
Change in mean Knee injury and Osteoarthritis Outcome Score subscale quality of life score [12,26]

The RCT included in this scrutiny assessed the heterogeneity among them and was characterized by limited sample sizes and disparate outcomes. Furthermore, after discussions with the reviewers (Y.K. and T.K.), it was concluded that the reliability of the outcome estimates was low. Consequently, the results were not pooled in the meta-analysis, and a non-integrated forest plot was presented instead (Figures 10-13) [20].

The GRADE evaluation compared MRT repair versus conservative therapy and conventional therapy versus multimodal therapy. The certainty of evidence for both comparisons was very low (Tables 4, 5).

**Table 4:** Summary of findings (conservative therapy compared to meniscus root tears repair for meniscus root tears) *The risk in the intervention group (and its 95% CI) is based on the assumed risk in the comparison group and the relative effect of the intervention (and its 95% CI). GRADE Working Group grades of evidence High certainty: we are very confident that the true effect lies close to that of the estimate of the effect. Moderate certainty: we are moderately confident in the effect estimate. The true effect is likely to be close to the estimate of the effect, but there is a possibility that it is substantially different. Low certainty: our confidence in the effect estimate is limited. The true effect may be substantially different from the estimate of the effect. Very low certainty: we have very little confidence in the effect estimate. The true effect is likely to be substantially different from the estimate of effect. Explanations a. It was downgraded by one level due to variations in effects b. Downgraded one level due to differences in intervention methods c. The grade was downgraded by one level due to the small sample size The symbols follow the GRADE system adopted by Cochrane to visually represent the quality of evidence. The combination of filled (⨁) and empty (◯) circles reflects the following evaluation: ⨁⨁⨁⨁: High quality ⨁⨁⨁◯: Moderate quality ⨁⨁◯◯: Low quality ⨁◯◯◯: Very low quality For detailed criteria, please refer to the official GRADE handbook: GRADE Handbook PDF (https://cgf.cochrane.org/sites/cgf.cochrane.org/files/uploads/uploads/how_to_grade.pdf?utm_source=chatgpt.com) ADL, activities of daily living; CI, confidence interval; GRADE, Grading of Recommendations Assessment, Development, and Evaluation; KOOS, Knee injury and Osteoarthritis Outcome Score; MD, mean difference; RCT, randomized controlled trial; QOL, quality of life

Outcomes	Anticipated absolute effects^*^ (95% CI)		Relative effect (95% CI)	No. of participants (studies)	Certainty of the evidence (GRADE)
	Risk with meniscus root tears repair	Risk with conservative therapy			
ADL assessed with KOOS subscale (scale from 0 to 100; mean follow-up: 4.2 years)	The mean ADL was 84.5 points	MD 9.1 points lower (22.09 lower to 3.89 higher)	-	48 (1 non-RCT)	⨁◯◯◯ Very low^a,b,c^
QOL assessed with KOOS subscale (scale from 0 to 100; mean follow-up: 4.2 years)	The mean QOL was 54.0 points	MD 0.5 points lower (18.74 lower to 17.74 higher)	-	48 (1 non-RCT)	⨁◯◯◯ Very low^a,b,c^

**Table 5:** Summary of findings (multimodal therapy compared to conventional therapy for meniscus root tears) *The risk in the intervention group (and its 95% CI) is based on the assumed risk in the comparison group and the relative effect of the intervention (and its 95% CI). GRADE Working Group grades of evidence High certainty: we are very confident that the true effect lies close to that of the estimate of the effect. Moderate certainty: we are moderately confident in the effect estimate. The true effect is likely to be close to the estimate of the effect, but there is a possibility that it is substantially different. Low certainty: our confidence in the effect estimate is limited. The true effect may be substantially different from the estimate of the effect. Very low certainty: we have very little confidence in the effect estimate. The true effect is likely to be substantially different from the estimate of effect. Explanations a. The grade was downgraded by two levels due to a high risk of bias associated with the blinding of participants b. It was lowered by one level due to variations in effects c. Downgraded one level due to differences in intervention methods d. The grade was downgraded by one level due to the small sample size The symbols follow the GRADE system adopted by Cochrane to visually represent the quality of evidence. The combination of filled (⨁) and empty (◯) circles reflects the following evaluation: ⨁⨁⨁⨁: High quality ⨁⨁⨁◯: Moderate quality ⨁⨁◯◯: Low quality ⨁◯◯◯: Very low quality For detailed criteria, please refer to the official GRADE handbook: GRADE Handbook PDF (https://cgf.cochrane.org/sites/cgf.cochrane.org/files/uploads/uploads/how_to_grade.pdf?utm_source=chatgpt.com) ADL, activities of daily living; CI, confidence interval; GRADE, Grading of Recommendations Assessment, Development, and Evaluation; KOOS, Knee injury and Osteoarthritis Outcome Score; MD, mean difference; RCTs, randomized controlled trials; QOL, quality of life

Outcomes	Anticipated absolute effects^*^ (95% CI)		Relative effect (95% CI)	No. of participants (studies)	Certainty of the evidence (GRADE)
	Risk with conventional therapy	Risk with multimodal therapy			
ADL assessed with KOOS subscale (scale from 0 to 100; mean follow-up: 4 months)	The mean ADL was 64.0 to 84.8 points	MD 1.70 to 2.78 points higher (5.74 lower to 6.69 higher)	-­	90 (2 RCTs)	⨁◯◯◯ Very low^a,b,c,d^
QOL assessed with KOOS subscale (scale from 0 to 100; mean follow-up: 4 months)	The mean QOL was 54.8 to 88.8 points	MD -1.00 to 1.78 points higher (13.51 lower to 6.66 higher)	-	90 (2 RCTs)	⨁◯◯◯ Very low^a,b,c,d^

Certainty of Evidence Comparing the Intervention Effects of MRT Repair and Conservative Therapy

In the comparison of intervention effectiveness between MRT repair versus conservative therapy, conservative therapy was associated with an MD of 9.1 points lower (95% CI= -22.09 to 3.89) on the KOOS subscale ADL and an MD of 0.5 points lower (95% CI= -18.74 to 17.74) in QOL. The certainty of evidence for the GRADE was very low (Table 4) [29].

Certainty of Evidence for Comparing the Intervention Effects of Conventional Versus Multimodal Therapy

In the comparison of intervention effectiveness between conventional therapy versus multimodal therapy, multimodal therapy was associated with an MD of 1.70 to 2.78 points higher (95% CI= -5.74 to 6.69) on the KOOS subscale ADL and an MD of -1.00 to 1.78 points higher (95% CI= -13.51 to 6.66) in QOL. The certainty of the evidence for this GRADE was very low (Table 5) [12,26].

Discussion

Summary of Results

This systematic review included 11 studies. The GRADE evaluation showed the following results: (i) in the comparison of intervention effectiveness between MRT repair versus conservative therapy, conservative therapy was associated with an MD of 9.1 points lower (95% CI= -22.09 to 3.89) on the KOOS subscale ADL and an MD of 0.5 points lower (95% CI= -18.74 to 17.74) in QOL, (ii) in the comparison of intervention effectiveness between conventional therapy versus multimodal therapy, multimodal therapy was associated with an MD of 1.70 to 2.78 points higher (95% CI= -5.74 to 6.69) on the KOOS subscale ADL and an MD of -1.00 to 1.78 points higher (95% CI= -13.51 to 6.66) in QOL, and (iii) the certainty of evidence was very low for both comparisons.

Therefore, the effectiveness of MRT repair and conservative therapy for patients with MRTs and the effectiveness of multimodal or adjunct therapy were not shown because of the risk of bias and low certainty of the evidence.

Interpretation and Comparison of Results

MRT repair versus conservative therapy: Both approaches improved the KOOS ADL and QOL subscales. Although conservative therapy has short-term benefits, its long-term effects plateau and may not persist. In contrast, MRT repair improved continuously over the long term, suggesting that it may offer long-term benefits. MRT repair is recommended as an initial intervention for MRTs [9]. Biomechanical evaluations have shown that MRT effectively re-establishes normal joint mechanics and contact pressures. Additionally, clinical studies evaluating patients who underwent root repair have demonstrated healing using second-look arthroscopy and magnetic resonance imaging [37]. However, 33.5% of patients who underwent MRT transitioned to TKA within 10 years [9]. Therefore, although MRT repair is an effective treatment, its long-term effectiveness must be evaluated over 10 years.

Conventional therapy versus multimodal or adjunct therapy: Both multimodal and adjunct therapies can improve the KOOS subscales ADL and QOL. However, the RCT studies reported for each treatment have short follow-up periods of less than six months, making it difficult to determine the effectiveness of any of the interventions [12,26,27]. Therefore, further RCTs are necessary to clarify the effects of conventional versus multimodal or adjunctive therapies.

Quality and Bias of Studies

Owing to concerns about the overall quality of the studies included in this systematic review, the results need to be interpreted with caution. All three RCTs had a high risk of bias [12,26,27]. In exercise therapy, it is often challenging to blind patients and evaluators to intervention studies. As the included RCTs involved exercise therapy interventions, they were not blinded and consequently had a high risk of bias.

Among the cohort studies, five of eight were rated as HQ [28,29,31,33,34], whereas three were rated as MQ [30,32,35]. HQ studies were of HQ because of the rigorous selection of participants, extended follow-up periods, and low dropout rates during the interventions. Conversely, MQ studies had potential bias risks related to participant selection, follow-up duration, and dropout rates [30,32,35]. Despite the differences in study design, cohort studies generally provided HQ evidence. Therefore, it is advisable to apply the findings of HQ studies in clinical practice. However, to improve the quality of future research, RCTs should be conducted with a low risk of bias.

Quality of the Evidence

In this systematic review, the quality of evidence was assessed using the GRADEpro guideline development tool. The results indicated that the certainty of the evidence for the intervention effects of MRT repair versus conservative therapy and conventional therapy versus multimodal therapy was very low. Reasons for the very low GRADE score for MRT repair versus conservative therapy include inadequate blinding of participants, variation in intervention methods and effectiveness, and small sample size. In addition, the comparison of conventional therapy versus multimodal therapy revealed variations in effectiveness, differences in intervention methods, and small sample sizes.

These factors indicate that the quality of the studies and the risk of bias affected the reliability of the results. Evaluating the quality and bias of studies is crucial for interpreting the results and applying them in clinical practice. It is essential to consider these factors when determining the most effective treatment. Improving the quality of future research, particularly through RCTs with a low risk of bias, is recommended.

Association of Outcome Results With Minimal Clinically Important Difference

Since GRADE was limited to specific outcomes, we considered the results of the KOOS subscales ADL and QOL, Lysholm knee scoring scale, and Tegner activity score, including the minimal clinically important difference (MCID) for each outcome.

The MCID for the KOOS is suggested to range from 8 to 10 points. Additionally, it has been reported as 9.5 points for the ADL subscale and 13.6 points or higher for the QOL subscale [38-40]. Both MRT repair [26,29] and conservative treatment [12,26] had exceeded the MCID in the short to medium terms, suggesting that both treatments may be beneficial. Additionally, the MCID for the Lysholm knee scoring scale was reported to be 10.3 points or more [38,39]. Both MRT repair [29] and conservative treatment [12,29,33,34] had exceeded the MCID in the short to medium terms, indicating the potential usefulness of both treatments. However, the 10-year incidence rates of osteoarthritis following meniscal repair, meniscectomy, and nonoperative treatment were 53.0%, 99.3%, and 95.1%, respectively, whereas the incidence rates following TKA were 33.5%, 51.5%, and 45.5%, respectively [9]. When comparing arthroscopic repair to partial meniscectomy or nonoperative treatment, MRT repair has been reported to have better outcomes at the 12-month follow-up [1]. From a long-term perspective, surgical treatment of MRTs is recommended at this stage.

Study Limitations

This systematic review had several limitations. First, although three RCTs [12,26,27] were included to address the low LoE reported in previous studies, the small sample size and variability in effects meant that the limitations of previous research could not be overcome. Second, various techniques were used for MRT repair and conservative treatment, which could lead to confounding factors. Third, the measured outcomes and follow-up periods should be interpreted cautiously because they are inconsistent across studies.

## Conclusions

It remains unclear which is more effective for ADL and QOL when comparing MRTs with conservative treatment, and conventional therapy with multimodal therapy or adjunct therapy. To influence future treatment decisions, long-term, high-quality research that includes both controlled and randomized trials with large sample sizes is necessary.

## Appendices

Appendix A: Newcastle Ottawa Quality Scale for cohort studies

Note: A study can be awarded a maximum of one star for each numbered item within the selection and outcome categories. A maximum of two stars can be given for comparability.

Selection

1) Representativeness of the exposed cohort

 a) Truly representative of the average _______________ (describe) in the community ★

 b) Somewhat representative of the average ______________ in the community ★

 c) Selected group of users, e.g., nurses, volunteers

 d) No description of the derivation of the cohort

2) Selection of the non-exposed cohort

 a) Drawn from the same community as the exposed cohort ★

 b) Drawn from a different source

 c) No description of the derivation of the non-exposed cohort

3) Ascertainment of exposure

 a) Secure record (e.g., surgical records) ★

 b) Structured interview ★

 c) Written self-report

 d) No description

4) Demonstration that outcome of interest was not present at the start of study

 a) Yes ★

 b) No

Comparability

1) Comparability of cohorts on the basis of the design or analysis

 a) Study controls for _____________ (select the most important factor) ★

 b) Study controls for any additional factor ★ (This criteria could be modified to indicate specific control for a second important factor.)

Outcome

1) Assessment of outcome

 a) Independent blind assessment ★

 b) Record linkage ★

 c) Self-report

 d) No description

2) Was follow-up long enough for outcomes to occur

 a) Yes (select an adequate follow-up period for outcome of interest) ★

 b) No

3) Adequacy of follow-up of cohorts

 a) Complete follow-up - all subjects accounted for ★

 b) Subjects lost to follow-up unlikely to introduce bias - small number lost - > ____ % (select an adequate %) follow-up, or description provided of those lost) ★

 c) Follow-up rate < ____% (select an adequate %) and no description of those lost

 d) No statement
